# Granular Cell Dermatofibroma: When Morphology Still Matters

**DOI:** 10.3390/dermatopathology8030041

**Published:** 2021-08-13

**Authors:** Gerardo Cazzato, Anna Colagrande, Antonietta Cimmino, Maricla Marrone, Alessandra Stellacci, Francesca Arezzo, Teresa Lettini, Leonardo Resta, Giuseppe Ingravallo

**Affiliations:** 1 Section of Pathology, Department of Emergency and Organ Transplantation (DETO), University of Bari Aldo Moro, 70124 Bari, Italy; anna.colagrande@gmail.com (A.C.); micasucci@inwind.it (A.C.); lettinit@yahoo.com (T.L.); leonardo.resta@uniba.it (L.R.); giuseppe.ingravallo@uniba.it (G.I.); 2 Section of Legal Medicine, Interdisciplinary Department of Medicine, Bari Policlinico Hospital, University of Bari Aldo Moro, 70124 Bari, Italy; mariclamarrone@hotmail.it (M.M.); alestellacci@gmail.com (A.S.); 3 Section of Ginecology and Obstetrics, Department of Biomedical Sciences and Human Oncology, University of Bari Aldo Moro, 70124 Bari, Italy; francesca.arezzo@uniba.it

**Keywords:** granular cell, dermatofibroma, skin, histiocytoma, immunohistochemistry

## Abstract

Dermatofibroma, also known as “fibrous histiocytoma”, is one of the most common cutaneous soft-tissue tumors. Many variants of dermatofibromas have been described, and knowledge of these variations is important to avoid misdiagnosis of a possibly more aggressive tumor. Histological features of different variants can coexist in the same lesion, but typical common fibrous histiocytoma features are generally found, at least focally, in all cases. However, when cellular changes make up the majority of the lesion, the histopathological diagnosis can become more complex and requires immunohistochemical investigations for correct nosographic classification. We present the case of a cutaneous fibrous histiocytoma, “granular cell” variant, found on the left leg of a 74-year-old woman.

## 1. Introduction

Dermatofibroma is a commonly occurring cutaneous entity usually centered within the dermis. Dermatofibromas are referred to as benign fibrous histiocytomas of the skin, superficial/cutaneous benign fibrous histiocytomas, or common fibrous histiocytomas. These mesenchymal cell lesions of the dermis clinically are firm subcutaneous nodules which occur on the extremities in the vast majority of cases and which may or may not be associated with overlying skin changes [1,2]. The pathological diagnosis is easy with a typical anamnestic background or a clear pathologic feature. However, many variants of dermatofibromas have been described [2], and knowledge of these variations is important to avoid misdiagnosis of a possibly more aggressive tumor. The main histologic variants are aneurysmal, hemosiderotic, cellular, epithelioid, atypical, lipidized, clear cell, palisading, atrophic, keloidal, granular cell, myxoid, lichenoid, balloon cell, and signet-ring cell variants [2,3,4]. Histological features of different variants can coexist in the same lesion [3] but typical common fibrous histiocytoma features are generally found, at least focally, in all cases [1,3]. The features of the variants may represent the predominant component of the lesion, making the identification of the histiocytoma more difficult [4]. Correct identification of these variants is important to avoid misdiagnosis of a possibly aggressive lesion. Furthermore, some variants have distinct clinical presentations and biological behavior, with different incidences of local recurrence and, in rare and controversial cases, metastasis, making correct diagnosis even more important [4,5].

## 2. Materials and Methods

**Figure 1:**
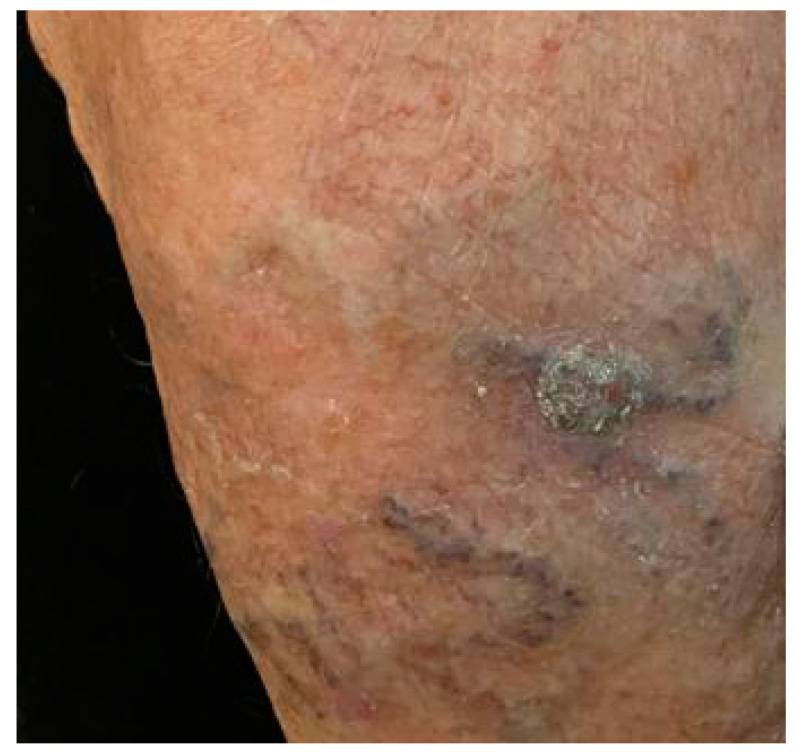
A grayish crusty and scaling plaque with a 30 mm of diameter was detected on the anterior middle third of the left leg.

A 74-year-old woman came to the attention of the Plastic Surgery Unit of the University Hospital of Bari with a dark-brown papule of her left leg, which appeared one year before and had slowly increased in size. She did not complain about pain or previous injuries in the region. A severe venous insufficiency of her legs was noticeable, with a superficial hypertrophic and congested venous capillary network. No other pathologies were referred to. The lesion was excised under local anesthesia and the residual loss of substance repaired with local flaps. At clinical examination a dark brown ovular ulcerated papule with a 30 mm of diameter was detected on the anterior middle third of the left leg (Figure 1). The lesion was retrieved in skin excision measuring 30 mm × 20 mm × 12 mm. No follow-up data are available. The lesion was fixed to the surrounding soft tissues, showing not well-defined margins. No macroscopic lymphadenopathies were found in the draining lymphatic fields. The tissue was formalin fixed and paraffin embedded, and 5 μm thick sections were prepared for hematoxylin and eosin (H&E) staining and immunohistochemical studies. Used antibodies included CD68 (Dako Denmark A/S, PG-M1, dilution 1:50), S-100 protein (Dako Denmark A/S, polyclonal, dilution 1:5000), CD34 antibody (AbCamEP373Y dilution 1:2500), Melan-A (Dako Denmark A/S, monoclonal, M7196, dilution 1:50), CD10 (Dako Denmark A/S, monoclonal, IS648, dilution 1:500), Ki-67 (Abcam, SP6, dilution 1:200).

## 3. Results

**Figure 2:**
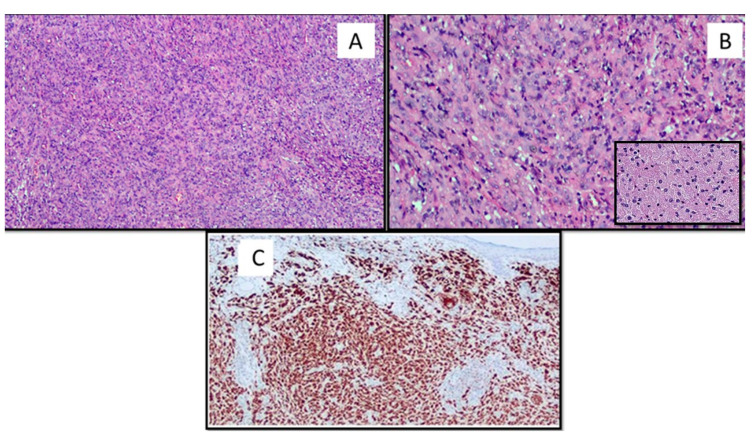
(**A**) The lesion was composed of a variable admixture of fibroblast-like cells and histiocytes that showed in more than 85% a large eosinophilic cytoplasm filled of granules or microvacuoles (hematoxylin and eosin, original magnification 40×). (**B**) Cytological details of the lesion with granular cytoplasm (original magnification 60×). Box: Histological details of the granular cytoplasm of the GCD constituent cells (original magnification 60×). (**C**) The neoplastic cells were strongly immunoreactive for CD68 (original magnification 40×).

The lesion occupied the derma and was composed of a variable admixture of fibroblast-like cells and histiocytes (Figure 2A). The latter were organized in large sheets and showed in more than 85% a large eosinophilic cytoplasm filled of granules or microvacuoles (Figure 2B). The interposed collagenous stroma was loose and rich in blood vessels. Inflammatory, predominantly lymphocytic, infiltrate, and globular collagen bundles were present in peripheral areas. The neoplasia presented defined margins with a pseudo capsule. The overlying epidermis was atrophic, with basal hyperpigmentation and melanin disposal. The lesion showed no mitotic activity.

Immunohistochemically, the cells expressed diffusely CD-68 (Figure 2C); S-100 protein was expressed only in Langerhans cells but was negative in the cells of interest; CD34, Melan-A, and CD10 expression were negative. The fraction of neoplastic proliferation was <1%, as assessed by Ki-67.

## 4. Discussion

Benign fibrous histiocytoma is considered as one of the most common benign tumors of the skin, with a very low recurrence rate ranging from 3% to 5% [5]. Our data are in agreement with the literature because dermatofibromas occur in people of all ages, although more commonly during the ages of 20 to 40, and develop more frequently in females than males, with as high as a 2:1 female to male predominance according to some reports. Aloi et al. [4] assert the benign nature of these lesions because described in literature was possible spontaneous regression and their relapses. The etiology is unknown, but some authors [3] recognize a possible etiopathogenetic cause to the local reaction of histiocytes after the local traumatic injury or an insect bite [5]. Dermatofibroma is clinically asymptomatic and painless. Macroscopically it is described as a roundish or ovoidal, firm dermal nodule, usually of less than 10 mm in diameter. It often shows a characteristic central white, scar-like patch on dermatoscopic examination [1,5].

Dermatofibroma with granular cells was described the first time in 1991 by LeBoit and Barr [3]. The cellular morphology of the lesion presents a large cytoplasm replete with coarse eosinophilic granules. The latter are due to an increased number of secondary lysosomes and maintain the same histiocyte cellular lineage [5]. Different theories [5,6] have been proposed to explain the mechanisms underlying lysosomal aggregation within the cytoplasm of these cells. It would appear that this process is caused by the dysfunction of a lysosomal enzyme or a lysosomal-associated protein involved in enzyme activation, rather than enzymatic targeting or lysosomal biogenesis [5,6]. These cytoplasmic changes are a constant features of granular cell tumors. However, cellular granularity can be observed in numerous cutaneous benign neoplasms and requires differential diagnosis elements (Table 1).

It is very important to consider the morphology and the immunohistochemical evaluation which, sometimes, can be decisive for the right diagnosis. Changes to granular cells can represent 30% to 90% of the entire tumor mass and it represents, as well as in our case, a great difficulty for the pathologist. Our case represents a difficult diagnosis because the tumor mass is made up of more than 85% of granular cells. Morphology of lesions could be a great help in differential diagnosis involving comparisons with the benign granular cell tumor (GCT), which differs from granular cell dermatofibroma (GCDF) by the different histogenesis: in fact, an immunohistochemical study with antibodies against the S-100 protein is strongly and diffusely positive in benign granular cell tumor, but negative in GCDF, just as CD68 will be positive in GCDF and almost totally negative in GCT [7,8].

Furthermore, the GCT appears to be positive for S-100 protein, CD68, CD63, and neuron-specific enolase, elements that can help in differential diagnostics with GCD.

Differential diagnosis with granular cell malignancy is apparently less complex because it has evident mitosis and cytological atypia [9,10], which could lead to the suspicion that it may be a malignant entity distinct from GCDF. Primary polypoid granular cell tumor (PPGCT) may represent another entity that must be correctly discriminated against by the GCDF; in more detail, PPGCT, although it can morphologically simulate GCDF, differs from it due to its strong and widespread positivity to the S-100 protein, which reflects its neural derivation. On the other hand, differential diagnosis is more complex with a subtype of PPGCT but which is not of neural derivation (“non neural”) [11]. In this case, the cells that make up this tumor are largely S-100 protein negative, and they turn out to be CD68 positive. However, this should not be misleading, as it is not a fibrohistocytic-derived tumor, but the positivity to CD68 is due to the accumulation of lysosomes that characterizes all neoplasms consisting, in fact, of granular cells. Therefore, the differential diagnosis can be made using the anti-NKI/C3 antibody which will intensely and homogeneously color the cells of the non-neural PPGCT, while it will be negative in the GCDF.

Simpler and more immediate is differential diagnosis with entities such as granular cell ameloblastoma (GCA) and granular cell basal cell carcinoma (GCBCC), which have very distinct morphological characteristics and which, even when the granular cells should represent the greatest part, allow detection of a certain percentage of typical neoplasm that helps the pathologist to make the correct diagnosis [12,13,14,15].

On the other hand, the granular cell variant of atypical fibroxanthoma is exceedingly rare, and in one study, granular cell changes were observed in 3 (1.8%) of 171 cases of AFX. Granular cell AFX stains negatively for S100 protein, making it easily distinguishable from conventional or malignant GCD. Workup and diagnosis of granular AFX is the same as the prototypical variant, and there does not appear to be any prognostic implications. Staining is positive for CD68 and vimentin and negative for Melan-A, human melanoma black (HMB)-45, S100 protein, pan-cytokeratin (CK), and actin [14,15].

Granular cell changes have also been observed in other neoplasm such as schwannoma, leiomyoma and leiomyosarcoma and the authors agree that correct morphological recognition together with the use of ancillary techniques (immunohistochemistry, IHC) are sufficient to make a correct diagnosis (smooth muscle actin, HHF35 actin, and desmin). On the other hand, the differential diagnosis of GCD with granular cell dermatofibrosarcoma protuberans (GCDFSP) is more complex [15].

It is a variant of the DFSP that undergoes granular cell changes, similarly to what we have previously described for other lesions. In this case, the morphological differential diagnosis may not be easy, although in the literature, there is a greater propensity of the DFSP to invade the subcutaneous tissue more than the DF (dermatofibroma). Nevertheless, in GCDFSP, in which the granular cell component is highly represented, it is mandatory to request IHC markers such as CD34, which is positive in GCDFSP and negative in GCDF [14,15]. It is very important to recognize this entity as the clinical and biological behavior is different compared to a typical DF.

In conclusion, granular cell dermatofibroma represents a rare histologic variant of dermatofibroma that is important to recognize because it can potentially be confused with other benign or malignant cutaneous neoplasms.

## Figures and Tables

**Table 1:** Neoplasm with granular cell components.

Benign granular cell tumor	Granular cell basal cell carcinoma
Malignant granular cell tumor	Granular cell schwannoma
Primitive polypoid granular cell tumor	Granular cell leiomyoma
Granular cell ameloblastoma	Granular cell leiomyosarcoma and angiosarcoma
Granular cell fibrous papule of the nose	Granular cell dermatofibrosarcoma protuberans
